# Statistical Reviewers Improve Reporting in Biomedical Articles: A Randomized Trial

**DOI:** 10.1371/journal.pone.0000332

**Published:** 2007-03-28

**Authors:** Erik Cobo, Albert Selva-O'Callagham, Josep-Maria Ribera, Francesc Cardellach, Ruth Dominguez, Miquel Vilardell

**Affiliations:** 1 Department of Statistics and Operations Research, Technological University of Catalonia, Barcelona, Spain; 2 Department of Internal Medicine, Vall d'Hebron Hospital, Barcelona, Spain; 3 Hematology Department, Hospital Universitari, Germans Trias i Pujol, Barcelona, Spain; 4 Departament de Medicina, Hospital Clínic i Provincial i Universitat de Barcelona, Barcelona, Spain; 5 Medicina Clínica, Barcelona, Spain; Johns Hopkins Bloomberg School of Public Health, United States of America

## Abstract

**Background:**

Although peer review is widely considered to be the most credible way of selecting manuscripts and improving the quality of accepted papers in scientific journals, there is little evidence to support its use. Our aim was to estimate the effects on manuscript quality of either adding a statistical peer reviewer or suggesting the use of checklists such as CONSORT or STARD to clinical reviewers or both.

**Methodology and Principal Findings:**

Interventions were defined as 1) the addition of a statistical reviewer to the clinical peer review process, and 2) suggesting reporting guidelines to reviewers; with “no statistical expert” and “no checklist” as controls. The two interventions were crossed in a 2×2 balanced factorial design including original research articles consecutively selected, between May 2004 and March 2005, by the Medicina Clinica (Barc) editorial committee. We randomized manuscripts to minimize differences in terms of baseline quality and type of study (intervention, longitudinal, cross-sectional, others). Sample-size calculations indicated that 100 papers provide an 80% power to test a 55% standardized difference. We specified the main outcome as the increment in quality of papers as measured on the Goodman Scale. Two blinded evaluators rated the quality of manuscripts at initial submission and final post peer review version. Of the 327 manuscripts submitted to the journal, 131 were accepted for further review, and 129 were randomized. Of those, 14 that were lost to follow-up showed no differences in initial quality to the followed-up papers. Hence, 115 were included in the main analysis, with 16 rejected for publication after peer review. 21 (18.3%) of the 115 included papers were interventions, 46 (40.0%) were longitudinal designs, 28 (24.3%) cross-sectional and 20 (17.4%) others. The 16 (13.9%) rejected papers had a significantly lower initial score on the overall Goodman scale than accepted papers (difference 15.0, 95% CI: 4.6–24.4). The effect of suggesting a guideline to the reviewers had no effect on change in overall quality as measured by the Goodman scale (0.9, 95% CI: −0.3–+2.1). The estimated effect of adding a statistical reviewer was 5.5 (95% CI: 4.3–6.7), showing a significant improvement in quality.

**Conclusions and Significance:**

This prospective randomized study shows the positive effect of adding a statistical reviewer to the field-expert peers in improving manuscript quality. We did not find a statistically significant positive effect by suggesting reviewers use reporting guidelines.

## Introduction

Despite being widely accepted as the best way to filter low-quality research, to detect flaws in scientific communications and to improve papers with significant contributions to their fields [1]–[3], peer review has also raised many criticisms [4]–[6]. Certainly, as a process carried out by humans it has its weaknesses and therefore many initiatives have been developed to improve it. For instance, some methodological mistakes are continually repeated in published papers and proposals that referees fail to detect or do not consider properly, and because of this, both the development of reporting guidelines [7]–[11] and the suggestion of adding methodological experts to the referees panel have been promoted [12] and implemented [13]. Accordingly, from what has been said, we might have expected the existence of strong scientific evidence in favor of peer review, but surprisingly, there have not been many attempts to determine “with the scientific rigor they demand of their authors” [14] its effect through measurable variables, and little evidence supports its use [15], [16]. Attempts to quantify its effects cannot deal with comparisons concerning the acceptance of reports without any intervention between submission and final publication. In fact, due to ethical and practical considerations, efforts have been specially made so as not to delay or interrupt the normal screening processes, so that the only alternative for evaluating the effects of peer review has been to assess surrogate variables.

The suggestion of adding a methodological expert to the reviewers pool of a Spanish biomedical journal gave us the chance to conduct a masked, randomized experiment to assess the effect of peer review, not only without interfering with the regular course of the editorial process, but also describing more realistically the true role of peer reviewing in improving the quality of papers. The two main objectives of our investigation were to assess the effects of (1) adding a statistical peer reviewer and (2) suggesting reporting guidelines [17]–[22] to reviewers, by taking into account a direct measurement of the final quality of papers instead of relying on surrogate variables.

## Methods

### Setting

Medicina Clínica (www.doyma.es/medicinaclinica) is a peer-reviewed weekly Spanish biomedical journal included in the Science Citation Index, the Current Contents, the Index Medicus and Excerpta Medica. It aims to publish original research papers, review articles, brief clinical notes, challenging editorials and the opinions of readers in the “letter to the editor” section. All submitted original research manuscripts are first evaluated by the journal's editorial committee, who decide which papers meet the journal's criteria and standards of relevance, and which are consequently sent for external peer-review, usually by two referees from the journal pool who are particularly familiar with the subject matter of the paper.

For this study, we included manuscripts sent to the “original” and “brief original” sections, which have to contain original primary research and include statistical analysis.

Qualitative research reports, case series, editorials, non-systematic reviews and letters to the editor were systematically excluded from the study.

### Data and allocation

Original research articles submitted consecutively to Medicina Clínica between May 2004 and March 2005 were assessed for eligibility (JMR, FC). Articles not fitting the journal's editorial policy were excluded.

We randomly allocated the manuscripts accepted for review into four groups defined by the interventions: Clinical reviewers (C) as normal procedure; Clinical reviewers plus a Statistical reviewer (CS); Clinical reviewers with checKlist (CK); and, Clinical reviewers plus a Statistical reviewer and checKlist (CSK). In this fashion, group C, acting as control group, only applied a clinical review, and therefore each article was sent to two clinical reviewers chosen from among the usual pool used by the journal. Papers were randomized once the two clinical peers had been chosen. Then, those allocated to the CS set were also sent to an expert statistical reviewer selected from the Medicina Clinica referee pool, which includes mainly senior methodological experts and graduate statisticians. A total of 39 methodological experts (table 1) were employed as statistical reviewers. Due to the late introduction, during the nineties, of formal statistical studies in Spain, reviewers with formal academic degrees in statistics were much younger (32.9 ( SD 2.7)) than statistical reviewers with academic degrees from applied bio-health disciplines. Although both authors and reviewers were warned that their material would be used to evaluate quality improvement during the editorial process, they were not warned about specific objectives.

**Table 1:** Statistical reviewers (n = 39) characteristics.

Academic degree	N (%)			
Technical	21 (53.8%)			
Statistics	16 (41.0%)			
Mathematics	3 (7.7%)			
Engineering	2 (5.1%)			
Applied	18 (46.2%)			
Medicine	13 (33.3%)			
Biology	3 (7.7%)			
Psychology	2 (5.1%)			
Current Employment	N (%)			
Universities	16 (41.0%)			
Non profit organization	16 (41.0%)			
Profit organizations	7 (17.9%)			
	Min	Max	Mean	SD
Age	29	64	41.5	9.1
Previous years reviewing MedClin (Barn)	2	10	5.0	1.5
Previous performed reviews	1	18	5.3	3.5

Manuscripts sorted into the CK intervention group were simply sent to the two clinical reviewers with a standard letter [“To facilitate your revision, you will find enclosed the reporting guideline from Bosch and Guardiola (Med Clin (Barc) 2003; 121:228–30). If you prefer, you may also employ one of the following documents: for clinical trials, the CONSORT statement (Ann Inter Med 2001;134:663–694); for meta-analysis, QUOROM (Lancet 1999; 354: 1896–1900); for diagnostic tests, STARD (Clinical Chemistry 2003;49:7–18); or the collections provided by the Scottish Intercollegiate Guidelines Network (http:/www.sign.ac.uk/guidelines/fulltext/50/annexc.html) or by Mora (Med Clin (Barc) 1999;113: 138–49”]. Reviewers were not asked to report whether they used the reporting guideline in reviewing the manuscript. Finally, manuscripts from the CSK group were defined by the use of both interventions.

Each paper was appraised (on a 9-point Likert scale) and classified (by EC) by study type (1: intervention, if a different treatment than standard was given to patients; 2: longitudinal, if observation lasted for more than one time point; 3: cross-sectional, just one time point observation; and 4-others, if the study could not fit into the previous groups, for example, non-human units, population data, meta-analyses and so on). Then, manuscripts were randomly allocated (by AS) using a computer program that first stratifies by study type, and second allocates to intervention groups while minimizing differences in initial quality. Then, the manuscripts followed the proper editorial procedure, according to their assigned group. See authors and reviewers agreement in supporting information file S1.

### Assessment and Procedure

A modified version of the Manuscript Quality Assessment Instrument (MQAI) designed by Goodman et al. (see supporting information file S2) [23] was used to assess the outcome. Each item assessed quality using a 5-point Likert scale from 1 (low) to 5 (high). Two specific items were added: one related to misconduct (item 1b), which includes suspicion of forgery, and one related to sample-size calculations (item 4b).

Two evaluators (EC, RD) independently rated the reporting quality of manuscripts at initial submission and following peer review and revision, according to the MQAI. Both knew the initial and final status but were blinded to the intervention group. The final score awarded to each scale item was reached by averaging the two evaluators' item scores after allowing each one to modify his or her score once the reasons for the other evaluator's score was made known. Primary outcome was defined as the difference in the quality of papers between the initial and final submission, expressed as the sum of the 36 specific MQAI items, resulting in a minimum of 36 (lowest quality) and a maximum of 180 points.

In order to evaluate the success of masking, the evaluators tried to guess which papers had been revised by a statistical reviewer (or with the help of a guideline) from the changes they observed in each article. The evaluators had to answer the following question by consensus: “Has it been reviewed by a statistician (or with the help of a guideline)?” The possible answers were “Yes”, “No” and “I don't know”. The blinding process was analyzed and considered successful if the evaluators' hit-proportion was not bigger than that expected by chance (50%). The cases with an answer “I don't know” were not included in the analysis. Only after the results had been validated and introduced into the database, was the group corresponding to each article revealed to the evaluators so that they could carry out the analysis.

### Study populations

Three different populations were considered: “complete” which included all randomized manuscripts not lost to follow-up, which was the population for the main analysis. Two other populations were defined for the sensitive analyses: one taking into consideration all “randomized” manuscripts and one including only those manuscripts accepted for publication. Those manuscripts rejected due to reviewers' comments and those lost to follow-up were analyzed considering two different values for their final quality: 1) the initial overall quality was imputed as the final overall quality interpreted as no change in quality during the editorial process), or 2) the final overall quality was assigned a value equal to the mean final quality value of the final versions of the received articles. The second method accounted for the positive effects of rejecting the low quality manuscripts, since the lower the initial score of the manuscript, the better the score in the initial-final difference assigned.

### Analysis and Sample Rationale

A 2×2 analysis of variance of the primary variable (change in quality score) was carried out to test the two hypotheses: 1, adding a statistical reviewer to the field expert peers and 2, suggesting reviewers reporting guidelines. Since both hypotheses addressed different objectives, no adjustment of the alpha risk consumption was made. Sample-size calculations indicated that a hundred articles were needed to reach an 80% power to detect a difference in means equivalent to a 55% of the intra-group standard deviation (α = 0.05; two-sided testing).

Secondary analysis were also based on ANOVA and included the same comparison of quality improvement by each individual item, as well as the segregated analysis of the rejected manuscripts and comparisons of initial quality between the originals that completed the editorial process and those which were lost to follow-up, by means of t-tests and χ^2^ tests. To check the effectiveness of the masking procedure, the percentage of matched trials between the appraisers' assessment and the real allocation of each article was computed. The main analysis was repeated stratifying by response to the masking question in order to analyze if it was able to account for the intervention effect.

## Results

### Enrolment and Randomization

**Figure 1:**
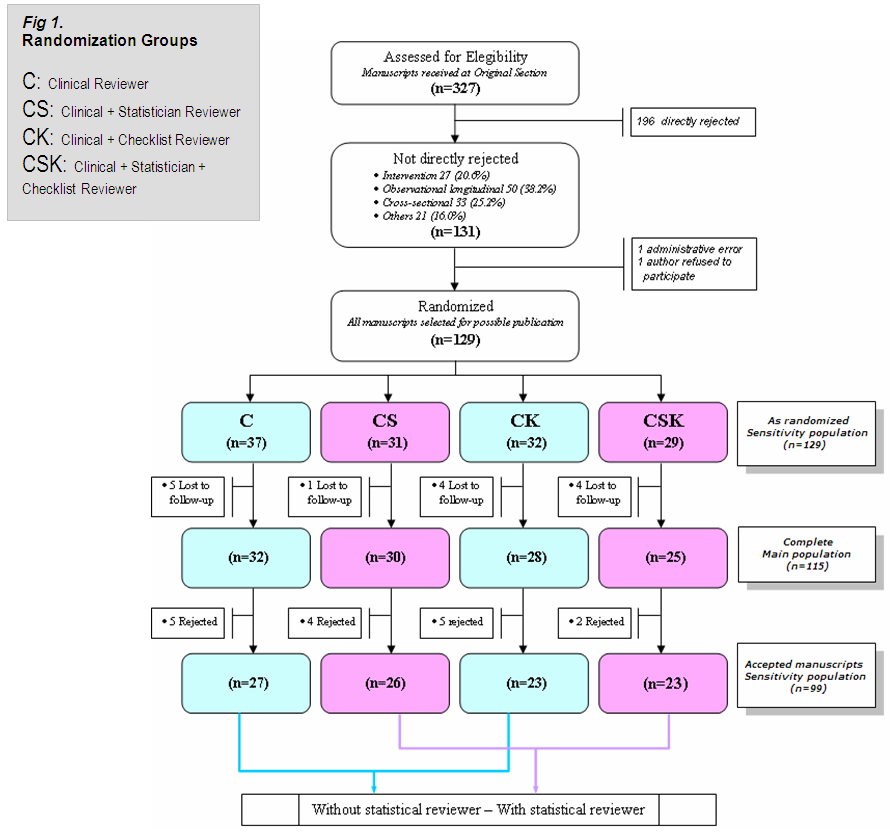
Flow chart of the review process among the four randomization groups. From 131 selected papers for possible publication, 129 were randomized and allocated into four comparison groups in a 2×2 factorial design to evaluate the effects of adding a statistical expert as a reviewer and of suggesting the use of checklists. Main analyses are based on the 115 papers which completed the review process.

Of the 327 originals received between May 2004 and March 2005, 196 (59.9%) were directly rejected by the editorial team. The remaining 131 (40.1%) were selected by the editorial committee as possible publications and therefore randomized. Of these, 2 were excluded either as a result of an administrative error (n = 1) or because the authors refused to participate (n = 1). From the 129 randomized manuscripts, 14 were lost of follow up because authors missed the deadline and the masked allocation was revealed; 21 (18.3%) of the 115 included papers were “interventions”, but only 3 were randomized clinical trials, 46 (40.0%) were longitudinal designs, 28 (24.3%) cross-sectional and 20 (17.4%) others. On the other hand, 16 were rejected by the editorial team after evaluating peer-review reports. The rejected papers had a significantly lower initial score on the overall Goodman scale than the accepted papers (difference 15.0, 95% CI from 4.6 to 24.4). No significant differences in initial quality were found between the lost to follow-up articles and the ones studied. Figure 1 shows the distribution of manuscripts among randomization groups.

### Descriptive Analysis of Initial Quality

The initial mean overall quality of the 115 originals analyzed was 84.5 (SD 19.1), without significant differences between the intervention groups. Table 2 shows baseline overall Goodman's scale by study type and allocated intervention group and Table 3 shows the baseline characteristics for each item by intervention group. In general, the manuscripts classified as “others”, followed by those reporting interventions, showed the lowest scores while, on average, longitudinal designs rated above the other types of study.

**Table 2:** Initial overall quality scores by study type, as assessed by two blinded evaluators on the Goodman scale.

INITIAL OVERALL QUALITY SCORES										
Group	C		CS		CK		CSK		ALL	
**Outcome**	**N**	**Mean**	**N**	**Mean**	**N**	**Mean**	**N**	**Mean**	**N**	**Mean**
TOTAL	32	81.0	30	81.0	28	88.8	25	88.2	115	84.5
*1. Intervention*	4	76,5	7	84,1	4	110,3	6	69,8	21	83,6
*2. Longitudinal*	14	84,6	10	86,5	12	86,9	10	93,6	46	87,6
*3. Cross-sectional*	9	85,9	8	71,5	6	93,0	5	90,2	28	84,1
*4. Other*	5	65,6	5	81,0	6	74,0	4	99,8	20	78,8

**Overall score is computed as the sum over the 36 individual items, each one rated from 1** (lowest) **to 5** (highest). **Minimum and maximum possible overall scale values were 36 and 180. Standard deviation was 19.**

**Table 3:** Mean initial individual quality scores assessed by two blinded evaluators on the Goodman scale (lowest = 1, highest = 5). Number of manuscripts is specified when that item did not apply to all manuscripts. Standard deviations ranged from 0.9 to 1.5.

INITIAL QUALITY SCORES												
Group			C		CS		CK		CSK		ALL	
G	1b	Lack of Oversight or Fake		3.3		3.3		3.6		3.6		3.5
G	1c	Organization		3.3		3.4		3.5		3.2		3.3
G	1d	Style		2.7		2.8		3.2		3.2		3.0
G	1e	Concise		2.9		2.6		3.0		2.8		2.8
I	2a	Background		3.0		3.4		3.7		3.6		3.4
I	2b	Aims		3.0		3.1		3.6		3.1		3.2
MM	3a	Setting and Source		3.3		2.9		3.2		3.2		3.1
MM	3b	Eligibility Criteria		2.9		3.0		3.0		2.9		3.0
MM	3c	Suitability of the comparison groups	24	2.0	21	2.3	18	2.5	18	2.7	81	2.3
MM	4a	Study Design		2.5		2.9		2.9	24	2.9	114	2.8
MM	4b	Sample size rationale		1.2	29	1.2	25	1.5	24	1.3	110	1.3
MM	4c	Masking	25	1.1	21	1.8	21	1.3	19	1.4	86	1.4
MM	5a	Major variables		2.9		2.9		3.0		3.4		3.0
MM	5b	Side-effects	12	1.8	13	1.6	7	3.1	12	2.3	44	2.1
R	6a	Lost to follow up		2.2	29	2.0	27	2.3		2.1	113	2.2
R	6b	Sample description		2.6		2.6		3.0		2.9		2.8
R	6c	Dropouts Description		2.5		2.6		2.9	24	2.6	114	2.6
R	7a	Quantitative Methods		2.3		2.4		2.5		2.6		2.4
R	7b	Clear Reporting		2.8		2.6		3.2		3.1		2.9
R	7c	Reporting of Denominators		2.9		3.3	27	3.3		3.5	114	3.2
R	7d	Effect Size	26	2.3	26	2.1	26	2.3	22	2.4	100	2.2
R	7e	Diagnostic tests	7	2.6	7	2.0	6	2.7	4	3.0	24	2.5
R	7f	Confidence Intervals		1.7		1.9		1.8		2.0		1.9
R	7g	Balanced between detail and summary		2.6		2.5		2.9		3.0		2.7
R	7h	Dropouts Analisys	24	1.5	19	1.7	16	1.4	15	1.5	74	1.5
R	7i	Analisys Multiple Measures	27	2.3	24	2.4	24	2.3	19	2.3	94	2.4
R	7j	Report Multiple Measures	20	2.3	16	2.5	17	2.4	15	2.4	68	2.4
R	7k	Subgroup Effects	31	2.5	29	2.5	27	2.4		2.5	112	2.5
R	7l	Figures&Tables		2.2		1.8		2.5		2.4		2.2
D	8a	New Knowledge in its field		2.0		1.9		2.4		2.4		2.2
D	8b	Other Supporting Evidence		2.5		2.8		2.9		2.9		2.8
D	8c	Limitations of the study		1.8		1.6		1.9		1.9		1.8
D	8d	Generalizing		2.1		1.8		2.3		2.4		2.1
D	8e	Strength and Tone		2.1		2.1		2.3		2.4		2.2
O	9a	Title		2.8		2.4		3.1		3.2		2.9
O	9b	Abstract	31	2.8	29	2.6		3.1		2.8	113	2.8

### Effects on the Primary Outcome

**Figure 2:**
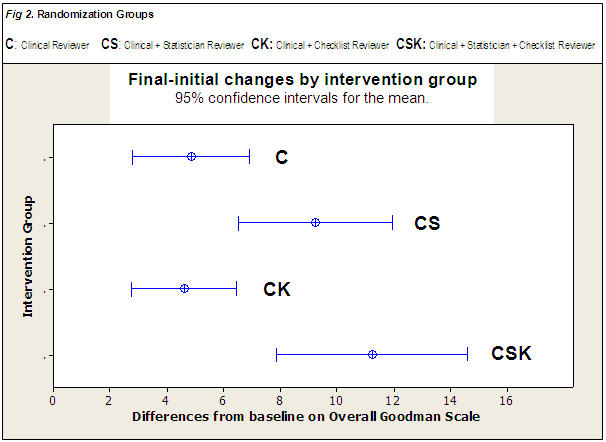
Mean quality changes, with uncertainty confidence intervals, from initial version to final version of the full Goodman scale. The overall quality is computed by the addition of the scores obtained in each one of the 36 items of the scale (first item is a summarizing measure of quality and thus, not included). The two groups without a statistical reviewer had a final-initial difference of 4.5 and 4.7 points, equivalent to a final-initial change in one item from very poor to excellent. Indeed, the two groups with a methodological referee changed 9.1 and 11.3, more than a complete reversal in the evaluation of two items. Thus, the effect size of adding a statistical reviewer is computed as the weighted average of the changes in groups CS+CSK minus changes in C+CK.

**Figure 3:**
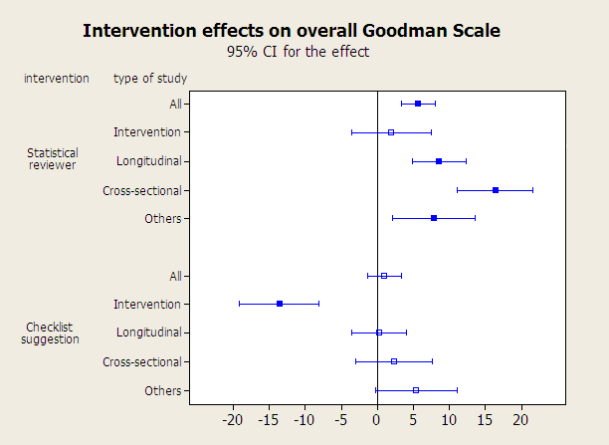
Intervention effects on overall Goodman Scale. Solid boxes indicate statistically significant effects.

Figure 2 shows confidence intervals for the change between initial and final values in the four randomized groups. The estimated effect (fig. 3) of adding a statistical reviewer was 5.5 (95% CI from 4.3 to 6.7) and the effect of sending a guideline to the authors was 0.9 (95% CI from −0.3 to +2.1) with no significant interaction effect between them (1.1, 95% CI from −0.1 to +2.3). Adding a statistical reviewer had a reasonably homogeneous effect among the four study types (fig. 3), but the suggestion of employing a checklist had a negative effect on the group of intervention papers. In the sensitive-analyses populations, we reached the same conclusions about those effects. Referring to the impact of non-complete data, the 14 papers lost to follow-up had a heterogeneous distribution among the randomized groups (figure 1) but did not differ, in terms of baseline quality, from the originals in the accepted manuscripts population. We performed several sensitivity analyses including those papers with different imputed values for the final version that produced very similar conclusions.

### Effects by each Individual Item

**Figure 4:**
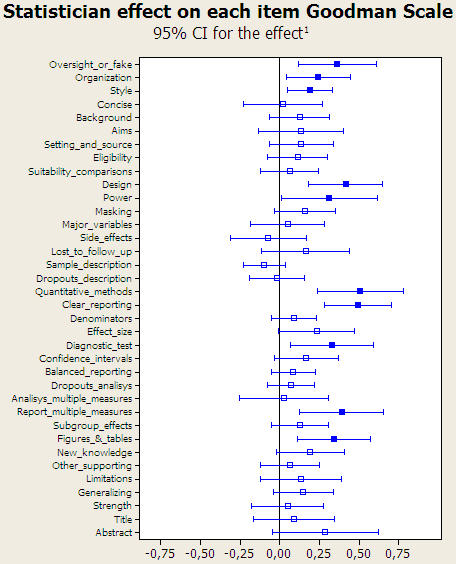
Estimated effect of statistical review over the 36 Goodman items. Solid boxes indicate statistically significant effects. ^1^ Differences in improvement from baseline between groups with and without statistical reviewer

**Figure 5:**
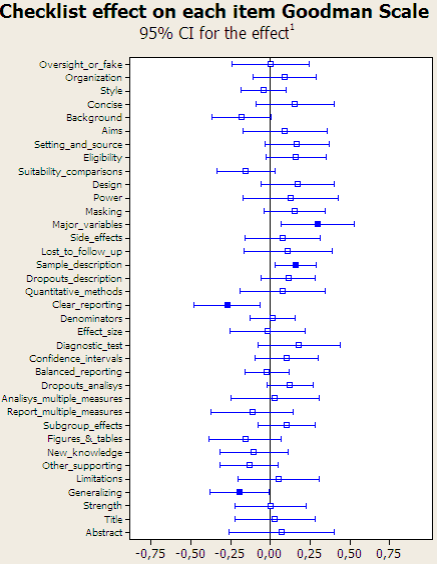
Estimated effect of checklist suggestion on the 36 Goodman items. Solid boxes indicate statistically significant effects. ^1^ Differences in improvement from baseline between groups with and without checklist suggestion

Figure 4 highlights the results for those items in which the effect of adding a statistician was found to be statistically significant. The by-item analysis showed a significant effect due to statistical review in 10 items. Individually, the items related to “quantitative methods” (effect size 0.50, [95% CI] from 0.23 to 0.77), “clear reporting” (0.49, from 0.27 to 0.70), “design” (0.41, from 0.17 to 0.64), and “reporting multiple measures” (0.38, from 0.12 to 0.65), were those where, on average, the statistical review most raised the quality of the submitted manuscript. Peer review by a statistician also had an impact on quality in items concerning the addition of ”oversight or fake”, “figures and tables”, “diagnostic tests”, “power”, “organization” and “style”. In contrast, the use of a recommended checklist by reviewers (Fig. 5) was positively significant in only 2 items; “major variables” and “sample description”, although 2 items had negative significant effects: “clear reporting” and “generalizing”.

### Successful Masking

On the “presence of a statistical reviewer” question, 20 (20.2%) originals were evaluated as “I don't know”. The remaining 79 originals (79.8%) were inspected with a match percentage of 60.8% (95% CI from 49.1 to 71.6%). On the use of a checklist, the evaluators were able to guess the intervention group in 65.3% (95% CI from 53.5 to 76.0%) of the 75 over 99 (75.8%) cases analyzed. The remaining 24 (24.2%) were evaluated as “I don't know”. After stratifying by response to both of the blinding questions, the overall conclusion on the main effects remained the same (statistical reviewer effect = 5.9, 95% CI = 3.4 to 8.5; checklist effect = −1.43, 95% CI = −3.8 to 0.99).

## Discussion

We have shown that the addition of a supplementary statistical reviewer improves manuscript quality during the editorial process. As the control intervention was “two clinical reviewers”, the estimated effect can be imputed either to the addition of an extra reviewer, or to the inclusion of a statistical expert, both of which confirm that peer review improves overall quality as measured by the MQAI. This result is consistently sustained by the alternative analysis of the sensitive populations. The guess (yes/no/don't know) of the statistician intervention was not able to remove the observed effect.

The size of the effect found can be interpreted in terms of specific item improvement: the 5.5 effect value counts as a 1-point quality improvement in a 5-point scale on more than five specific items. Although this effect is significant and positive, its size is very small related to the scale range (3.8%) but medium size (85.9%) related to the improvement variability (6.4), which may be due to some prudence or cautiousness during masked evaluation. In any case, in the light of the two evaluator scores, there is still room for improvement.

Mainly because it is not easy to check peer review without interfering with the editorial process, but also because it is considered a self-evident idea, the scientific testing of a process that is essential for science, which filters and shapes scientific communications and decides major research funding, has barely deserved the interest of researchers. Some research groups have tried to assess the effect on the quality of peer review of training evaluators [24], blinding and unmasking [25]–[27], referee characteristics and publication language [28], and feedback by editors [29]. Some assessed the blinding effect on the acceptance of papers, either in randomized [30] or non-randomized comparisons [31]. Others analyzed, in a historical cohort, the report of “positive” findings [32]. Schriger et al studied the changes after peer review and editing in tables and figures in a cohort of 62 randomized clinical trials submitted to BMJ [33]. However, only the randomized retrospective evaluation of Goodman et al [23] and the paired comparison of Pierie et al [34] measured reporting quality. A systematic review undertaken in 2002 concluded “Peer review, although widely used, is largely untested and its effects are uncertain” [35]. Our searches in Medline found only one (although non-randomized) study, suggesting that a statistical checklist could improve report quality [36]. Surprisingly, very few studies have analyzed the true outcome of peer review: manuscript quality instead of review quality. Although it is possible to think of further indicators of research quality, perhaps related to positive impact [37], the MQAI [23] has the advantage of being the only scale developed out of a randomized study that measures reporting quality and can be applied to a broad set of studies. As far as we know, this is the first prospective randomized trial assessing the effect of peer review to have a positive result. In 2001 we carried out a similar study on 43 manuscripts to estimate the effect of reviewers, which was significant in several secondary variables, but not in the principal, although this did show a trend towards a positive effect [38]. Those results encouraged us to extensively review our design and methods. Basically, what we have added to this new study is a complete follow-up, including those manuscripts finally rejected, with the analysis of alternative sensitive analyses.

The 14 papers lost to follow-up did not differ, in terms of baseline quality, from the originals in the complete population. On the other hand, the 16 rejected papers present a significantly lower initial score on the overall Goodman scale than the 99 accepted papers. However, we have to be careful when interpreting these results as the two evaluators, although blinded to the intervention group, knew the editorial decision-as they didn't have the final manuscript version.

For this study we have concentrated on the quantitative results. We do not provide qualitative information about what the statistical reviewers actually did to improve the manuscripts or how they differ from clinical reviewers. Furthermore, we did not study if authors followed all of the reviewer's suggestions, either clinical or methodological; or if there are manuscript characteristics related to potential improvement introduced by peer review.

It should also be stressed that our target population was a single journal with an impact factor of just over 1: external validity of our results may be compromised if the positive effect of including a methodological reviewer depends upon journal, paper or reviewer characteristics. If journals with higher impact factors have better methodological papers [39], their room for improvement may be lower. But on the other hand, it could also be considered that those journals may also have better methodological reviewers.

We did not find statistical significance on the effect of enclosing a Spanish checklist [17] and suggesting English reporting guidelines such us CONSORT or STARD to Spanish reviewers. Unfortunately, because referees were not asked to return the completed checklists, we are not able to determine if they employed the proper guide for the study reviewed or if they misused the guide. It is very difficult to interpret the negative result in the intervention subgroup. In terms of alpha risk consumption and regression to the mean (since baseline quality was higher for group 3) both may lead to an erroneous conclusion, but as very few studies were randomized clinical trials and most of them were before-after studies, without well-known guidelines, a negative effect may have been produced. In any event, the fact that the evaluators were able to guess the presence of the reporting guideline in 65.3% (95% CI: 53.5 to 76.0%) of papers suggests the need for a new trial with an improvement in checklist delivery and feedback.

Here we have shown scientific evidence that peer review has a positive effect on the final quality of papers, by means of demonstrating, in a randomized trial with masked evaluation, the effects of adding a methodological expert to the review panel. Nonetheless, there is still a long way to go to ensure that scientific communications achieve the maximum quality. Even, if peer review is not the last system to improve research or, at least, to improve scientific journals and reporting [40].

## Supporting Information

Text S1 Authors and reviewers agreement
(0.02 MB DOC)

Text S2 Goodman scale
(0.11 MB DOC)

## References

[1] Editorial (2000). Peering into the review process. Nat Struct Biol.

[2] Scarpa T (2006). Peer Review at NIH.. Science.

[3] Rennie D (1992). Editorial peer review: let us put it on trial.. Controlled Clinical Trials.

[4] Editorial (2001). Bad peer reviewers.. Nature.

[5] Lock S (1994). Does Editorial Peer Review Work?. Ann Intern Med.

[6] Hanks G (2005). Peer review in action: the contribution of referees to advancing reliable knowledge.. Palliat Med.

[7] Begg C, Cho M, Eastwood S, Horton R, Moher D (1996). Improving the quality of reporting of randomized controlled trials. The CONSORT statement.. JAMA.

[8] Moher D, Schulz KF, Altman DG, CONSORT Group (2001). The CONSORT statement: revised recommendations for improving the quality of reports of parallel-group randomized trials.. JAMA.

[9] Campbell MK, Elbourne DR, Altman DG, CONSORT Group (2004). The CONSORT statement: extension to cluster randomized trials.. BMJ.

[10] Des Jarlais DC, Lyles C, Crepaz N, the TREND Group (2004). Improving the reporting quality of nonrandomized evaluations of behavioral and public health interventions: the TREND Statement.. American Journal of Public Health.

[11] STROBE group STROBE statement [homepage on internet]. Strengthening the Reporting OBservational studies in Epidemiology [updated 12 june 2006; cited 20 October 2006 ]..

[12] Altman DG (2002). Poor-quality medical research. What can journals do?. JAMA.

[13] Gore SM, Jones G, Thompson SG (1992). The Lancet's statistical review process: areas for improvement by authors.. Lancet.

[14] Enserink M (2001). Scientific publishing. Peer review and quality: a dubious connection?. Science.

[15] (2002). The fourth international congress on biomedical peer review. JAMA.

[16] Altman D, Schulz KF, Godlee F, Jefferson T (1999). Statistical peer review.. Peer review in health sciences.

[17] Bosch F, Guardiola E, Esteve Foundation Workshop 2002 group (2003). Lista de comprobación (checklist) abreviada e evaluación de artículos de investigación biomédica básica.. Med Clin (Barc).

[18] Altman DG, Schulz KF, Moher D, Egger M, Davidoff F (2001). The Revised CONSORT Statement for Reporting Randomized Trials: Explanation and Elaboration.. Ann Intern Med.

[19] Moher D, Cook D, Eastwood S, Olkin I, Rennie D (1999). Improving the quality of reports of meta-analyses of randomised controlled trials: the QUOROM statement.. Lancet.

[20] Bossuyt PM, Reitsma JB, Bruns DE, Gatsonis CA, Glasziou PP (2003). The STARD Statement for Reporting Studies of Diagnostic Accuracy: Explanation and Elaboration.. Clinical Chemistry.

[21] Scottish Intercollegiate Guidelines Network [homepage on internet; cited 5 february 2007]..

[22] Mora-Ripoll M (1999). Cómo mejorar la calidad estadística de los artículos presentados a revistas biomédicas: lista de comprobación para los autores.. Med Clin (Barc).

[23] Goodman SN, Berlin J, Fletcher SW, Fletcher RH (1994). Manuscript quality before and after peer review and editing at Annals of Internal Medicine.. Ann Intern Med.

[24] Schroter S, Black N, Evans S, Carpenter J, Godlee F (2004). Effects of training on quality of peer review: randomized controlled trial.. BMJ.

[25] van Rooyen S, Godlee F, Evans S, Smith R, Black N (1998). Effect of blinding and unmasking on the quality of peer review: a randomized trial.. JAMA.

[26] Godlee F, Gale CR, Martyn CN (1998). Effect on the quality of peer review of blinding reviewers and asking them to sign their reports: a randomized controlled trial.. JAMA.

[27] van Rooyen S, Godlee F, Evans S, Black N, Smith R (1999). Effect of open peer review on quality of reviews and on reviewers' recommendations: a randomized trial.. BMJ.

[28] Nylenna M, Riis P, Karlsson Y (1994). Multiple blind reviews of the same two manuscripts. Effects of referee characteristics and publication language.. JAMA.

[29] Callaham ML, Knopp RK, Gallagher EJ (2001). Effect of written feedback by editors on quality of reviews: two randomized trials.. JAMA.

[30] Smith J, Nixon R, Bueschen AJ, Venable DD, Henry HH (2002). Impact of blind versus unblind abstract review on scientific program content.. J Urol.

[31] Ross J, Gross G, Desai M, Hong Y, Grant A (2006). Effect of blinded peer review on abstract acceptance.. JAMA.

[32] Ridker P, Torres J (2006). Reported outcomes in major cardiovascular clinical trials funded by for-profit and not-for-profit organizations: 2000-2005.. JAMA.

[33] Schriger DL, Sinha R, Schroter S, Liu PY, Altman DG (2006). From submission to publication: a retrospective review of the tables and figures in a cohort of randomized controlled trials submitted to the British Medical Journal.. Ann Emerg Med.

[34] Pierie JP, Walvoort H, Overbeke J (1996). Reader's evaluation of peer review and editing on quality of articles in the Nederlands Tijdschrift voor Geneeskunde.. Lancet.

[35] Jefferson T, Alderson P, Wager E, Davidoff F (2002). Effects of editorial peer review: a systematic review.. JAMA.

[36] Gardner MJ, Bond J (1990). An exploratory study of statistical assessment of papers published in the British Medical Journal.. JAMA.

[37] Porta M, Fernandez E, Bolúmar F (2006). Commentary: the ‘bibliographic impact factor’ and the still uncharted sociology of epidemiology.. Int J Epidemiol.

[38] Arnau C, Cobo E, Ribera JM, Cardellach F, Selva A (2003). Effect of statistical review on manuscript quality in Medicina Clinica (Barcelona): a randomized study.. Med Clin (Barc).

[39] Lee K, Schotland M, Bachetti P, Bero L (2002). Association of journal quality indicators with methodological quality of clinical research articles.. JAMA.

[40] Campanario JM (1998). Peer review for journals as it stands today-Part 2.. Science Communication.

