# Supplementary material for: Statistical Reviewers Improve Reporting in Biomedical Articles: A Randomized Trial
Source: PLoS One. 2007 Mar 28;2(3):e332. doi: 10.1371/journal.pone.0000332 (PMC1824709; doi:10.1371/journal.pone.0000332)
Supplement: Text S1 — Authors and reviewers agreement (0.02 MB DOC) [file pone.0000332.s001.doc]

Authors and reviewers agreement

Both authors and reviewers were informed, but no research ethics committee was involved.

Letter to the reviewers stated:

“*Medicina Clínica is running an improvement editorial quality program which implies monitoring your revision, always taking into account your confidentiality rights*”.

Letter to authors stated:

“*Medicina Clínica is running an improvement editorial quality program which implies monitoring your manuscript, always taking into account your confidentiality rights. Please, inform us if you wish to be excluded from it*.”
