# Supplementary material for: Statistical Reviewers Improve Reporting in Biomedical Articles: A Randomized Trial
Source: PLoS One. 2007 Mar 28;2(3):e332. doi: 10.1371/journal.pone.0000332 (PMC1824709; doi:10.1371/journal.pone.0000332)
Supplement: Text S2 — Goodman scale (0.11 MB DOC) [file pone.0000332.s002.doc]

**ANNEX.** Manuscript Quality Assessment Instrument designed by Goodman et al. (MQAI). N. Goodman, J. Berlin, S. W. Fletcher, R. H. Fletcher, *Ann Intern Med*. **121**, 11 (1994).

| Manuscript number: | Study type: 1 intervention 2 longitudinal 3 cross-sectional 4 other | | | |
| --- | --- | --- | --- | --- |
| Evaluator | | Date 1^st^ review | | Date 2^nd^ review |
| 1.- *General Evaluation* | | | | |
| a. *How would you describe the* ***overall quality*** *of this report?* | | | | |
| *1 Version* 1 2 3 4 5 6 7 8 9 | | | *2 Version* 1 2 3 4 5 6 7 8 9 | |
| b. *Do you think there is some kind of fake?* ***Oversight or fake*** | | | | |
| *1 Version* 1 2 3 4 5 NA | | | *2 Version* 1 2 3 4 5 NA | |
| c. *How good is the organization of this report? For example, are all methods in the methods section, all results in the results section?* ***Organization*** | | | | |
| *1 Version* 1 2 3 4 5 NA | | | *2 Version* 1 2 3 4 5 NA | |
| d. *How would you describe the style of the presentation?* ***Style*** | | | | |
| *1 Version* 1 2 3 4 5 NA | | | *2 Version* 1 2 3 4 5 NA | |
| e. *Is the manuscript concise?* ***Concise*** | | | | |
| *1 Version* 1 2 3 4 5 NA | | | *2 Version* 1 2 3 4 5 NA | |
| 2.- *Introduction* | | | | |
| a. *How clear are the background and rationale for this study*? (*The frequency and severity of the clinical problem, what remains unknown about the research question, and how patients could benefit from the answer*.) ***Background*** | | | | |
| *1 Version* 1 2 3 4 5 NA | | | *2 Version* 1 2 3 4 5 NA | |
| b. *How clear are the specific aims of this study?* *(The research questions, distinguishing main from secondary, and, if appropriate, hypotheses about what will be found)* ***Aims*** | | | | |
| *1 Version* 1 2 3 4 5 NA | | | *2 Version* 1 2 3 4 5 NA | |
| 3.- *Methods: Subjects* | | | | |
| a. *How adequate is the description of the setting of the study and source of subjects? To help readers understand whether the patients in the study are like theirs, the manuscript should provide information on when and where the research took place, a description of the level of care (community, primary care, referred), and if patients were referred, the pattern (source, distance, route) of referral.* ***Setting_and_source*** | | | | |
| *1 Version* 1 2 3 4 5 NA | | | *2 Version* 1 2 3 4 5 NA | |
| b. *How clear are the eligibility (inclusion and exclusion) criteria? Is diagnostic well defined?* ***Eligibility*** | | | | |
| *1 Version* 1 2 3 4 5 NA | | | *2 Version* 1 2 3 4 5 NA | |
| c. *For studies in which two groups are compared, is there enough information to judge the suitability of the comparison groups? How well was it reported how patients were chosen (for observational studies) or allocated (for experiments) so that readers can judge whether the researchers have compared like with like* ***Suitability comparisons*** | | | | |
| *1 Version* 1 2 3 4 5 NA | | | *2 Version* 1 2 3 4 5 NA | |
| 4. *Methods: Design* | | | | |
| a. *How clear is the study design? Do you understand what the authors set out to do and how they did it (the study design)? Are the treatments well defined?* ***Design*** | | | | |
| *1 Version* 1 2 3 4 5 NA | | | *2 Version* 1 2 3 4 5 NA | |
| b. *How clear is the sample size rationale and study power?* ***Power*** | | | | |
| *1 Version* 1 2 3 4 5 NA | | | *2 Version* 1 2 3 4 5 NA | |
| c. *How adequate is the description of the masking (i.e. blinding) procedure? Is it clear who was blinded, what blinding procedure was used, and the degree to which blinding was achieved* ***Masking*** | | | | |
| *1 Version* 1 2 3 4 5 NA | | | *2 Version* 1 2 3 4 5 NA | |
| 5. *Methods: Variable measurement* | | | | |
| a. *Is the operational definition of major variables clear enough so their strengths and limitations can be assessed? For example. in surveys, case definitions; in cohort studies, definitions for exposure and disease status; in diagnostic studies, the test procedure; for case control studies. Is it clear how cases and controls were defined? Other major variables might include important confounders, compliance, etc.* ***Major variables*** | | | | |
| *1 Version* 1 2 3 4 5 NA | | | *2 Version* 1 2 3 4 5 NA | |
| b. *How adequate is the reporting of important side-effects? For example, what are the types and numbers?* ***Side effects*** | | | | |
| *1 Version* 1 2 3 4 5 NA | | | *2 Version* 1 2 3 4 5 NA | |
| 6. *Results: Subjects* | | | | |
| a. *How complete is the information (reasons and numbers) on eligible subjects who were not included? For example, subjects might not be included because they refused to participate, their records were lost, or they were not compliant during a run-in period. Is there enough information to judge, even in a general way, the comparability of the participants and non-participants in the study?* ***Lost to follow up*** | | | | |
| *1 Version* 1 2 3 4 5 NA | | | *2 Version* 1 2 3 4 5 NA | |
| b. *How adequate is the description of the enrolled sample, including potential cofounders, effect modifiers, co-interventions, comorbidities and spectrum of disease? (In comparative studies, this would mean description by group).Is there a description ( a table when necessary ) of the characteristics of the enrolled sample, including potentially important demographic and prognostic factors or other descriptors that would help you to evaluate the comparability of the groups and/or the generalizability of the study results?* ***Sample description*** | | | | |
| *1 Version* 1 2 3 4 5 NA | | | *2 Version* 1 2 3 4 5 NA | |
| c. *How clear are the outcomes for everyone enrolled in the study? In addition to the main outcomes of the study, how well do the authors document the number of protocol violations, dropouts, crossovers, subjects with incomplete data, subjects who died for reasons other than the main reason under study, etc?* *(dropouts)* ***Dropouts description*** | | | | |
| *1 Version* 1 2 3 4 5 NA | | | *2 Version* 1 2 3 4 5 NA | |
| 7. *Results: Quantitative Reporting* | | | | |
| a. *Are the quantitative methods the right ones for the research questions and data? Are they referenced? Are the methods appropriate for the unit of analysis (e.g., person, events, or clusters), sample size and type of outcome (e.g., dichotomous or continuous. time to event* ***Quantitative methods*** | | | | |
| *1 Version* 1 2 3 4 5 NA | | | *2 Version* 1 2 3 4 5 NA | |
| b. *Are quantitative results reported in a manner that most of the intended audience could understand? Consider whether units are clear (particularly of regression coefficients), whether the results are in the most accessible scale (e.g., non-logarithmic), and whether there should be additional effort to interpret technical results for the reader* ***Clear reporting*** | | | | |
| *1 Version* 1 2 3 4 5 NA | | | *2 Version* 1 2 3 4 5 NA | |
| c. *How adequate is the reporting of denominators? For averages, percentages, rates, ratios, etc.* ***Denominators*** | | | | |
| *1 Version* 1 2 3 4 5 NA | | | *2 Version* 1 2 3 4 5 NA | |
| d. *Are the magnitudes of effects reported? "Effects" include odds ratios, risk differences, differences between means, regression coefficients, etc. (but not P values), and should be either stated directly or readily apparent from the data presented* ***Effect size*** | | | | |
| *1 Version* 1 2 3 4 5 NA | | | *2 Version* 1 2 3 4 5 NA | |
| e. *In studies of diagnostic tests, how adequate is the reporting of summary statistics for test performance? Summary, statistics include sensitivity, specificity, predictive value. ROC curve. or likelihood ratio* ***Diagnostic test*** | | | | |
| *1 Version* 1 2 3 4 5 NA | | | *2 Version* 1 2 3 4 5 NA | |
| f. *Are confidence intervals or standard errors reported for main outcomes? If the main outcome is a difference between groups. or within patients, the statistical precision of that difference should be reported.* ***Confidence intervals*** | | | | |
| *1 Version* 1 2 3 4 5 NA | | | *2 Version* 1 2 3 4 5 NA | |
| g. *How appropriate is the balance between detail and summary results?* ***Balanced reporting*** | | | | |
| *1 Version* 1 2 3 4 5 NA | | | *2 Version* 1 2 3 4 5 NA | |
| h.? *How appropriately are dropouts, crossovers, or subjects with incomplete data dealt with in the analysis? Techniques to deal with these problems include intention-to-treat analyses, comparison of these groups at baseline, analyses stratified by these factors. and sensitivity analyses* ***Dropouts analysis*** | | | | |
| *1 Version* 1 2 3 4 5 NA | | | *2 Version* 1 2 3 4 5 NA | |
| i. *How adequate is the method used to control or assess the effects of multiple measured variables? If multiple variables are considered only singly, should joint effects be evaluated? Is a reasonable multivariate method chosen (e.g. stratification. adjustment, regression, ANOVA)? Does the variable coding permit adequate control?* ***Analysis multiple measures*** | | | | |
| *1 Version* 1 2 3 4 5 NA | | | *2 Version* 1 2 3 4 5 NA | |
| j. *How adequate is the reporting of analyses of multiple variables? Are we told how the initial pool of possible predictors was chosen, how the final ones were selected, the coefficients or effects (in interpretable units) of all terms in the final model, the coding of each variable, and the number of subjects with each predictor or the spread of predictor variables?* ***Report multiple measures*** | | | | |
| *1 Version* 1 2 3 4 5 NA | | | *2 Version* 1 2 3 4 5 NA | |
| k. *Are clinically relevant subgroup effects explored in appropriate detail (neither too much nor too little)?* ***Subgroup effects*** | | | | |
| *1 Version* 1 2 3 4 5 NA | | | *2 Version* 1 2 3 4 5 NA | |
| l. *Do the figures and tables effectively summarize important data? Include in your judgment whether tables and figures are accurate and clear; whether tabular data would be better presented graphically or vice-versa, and whether the balance between text and figures/tables is appropriate* ***Figures & tables*** | | | | |
| *1 Version* 1 2 3 4 5 NA | | | *2 Version* 1 2 3 4 5 NA | |
| 8. *Discussion and Conclusions* | | | | |
| a. *Is it clear what this study adds to the body of knowledge in its field?* ***New knowledge*** | | | | |
| *1 Version* 1 2 3 4 5 NA | | | *2 Version* 1 2 3 4 5 NA | |
| b. *How appropriate is the presentation of other supporting evidence that may be relevant to these conclusions (including theoretical reasoning, basic science results)? An appropriate presentation would be neither too detailed nor deficient.* ***Other supporting*** | | | | |
| *1 Version* 1 2 3 4 5 NA | | | *2 Version* 1 2 3 4 5 NA | |
| c. *How appropriate is the discussion of study limitations? An appropriate discussion would be neither too detailed nor deficient* ***Limitations*** | | | | |
| *1 Version* 1 2 3 4 5 NA | | | *2 Version* 1 2 3 4 5 NA | |
| d. *Is it clear if the authors are generalizing? If so are these generalizations justified?* ***Generalizing*** | | | | |
| *1 Version* 1 2 3 4 5 NA | | | *2 Version* 1 2 3 4 5 NA | |
| e. *Is the strength and/or tone of the conclusions appropriate to the design and results?* ***Strength*** | | | | |
| *1 Version* 1 2 3 4 5 NA | | | *2 Version* 1 2 3 4 5 NA | |
| 9. *Title and abstract* | | | | |
| a. *How good is the title? For example, clear, concise, and accurate Title* | | | | |
| *1 Version* 1 2 3 4 5 NA | | | *2 Version* 1 2 3 4 5 NA | |
| b. *Does the abstract adequately summarize the data and conclusions?* ***Abstract*** | | | | |
| *1 Version* 1 2 3 4 5 NA | | | *2 Version* 1 2 3 4 5 NA | |
